# Cell‐Type‐Specific HSPA1B Expression Links COPD and Atherosclerosis

**DOI:** 10.1002/iid3.70530

**Published:** 2026-09-29

**Authors:** Yangxin Zhao, Fangzheng Cao, Houwen Zhang, Yu Liang, You Wu

**Affiliations:** ^1^ Department of Neurology The Second Affiliated Hospital of Zhejiang Chinese Medical University Hangzhou China; ^2^ The Second Clinical Medical College of Zhejiang Chinese Medical University Hangzhou China

## Abstract

**Background:**

Chronic obstructive pulmonary disease (COPD) and atherosclerosis frequently coexist, but their shared immune mechanisms remain incompletely understood.

**Methods:**

We integrated single‐cell RNA‐sequencing datasets from COPD lungs and atherosclerotic lesions to characterize immune‐cell composition, intercellular communication, and overlapping upregulated genes. Cell‐type‐specific sc‐eQTL data from oneK1K were further combined with FinnGen GWAS summary statistics for COPD and coronary atherosclerosis using Mendelian randomization.

**Results:**

Both diseases showed a macrophage/monocyte‐centered immune architecture, with macrophages serving as major communication hubs. Among overlapping genes evaluated genetically, HSPA1B showed consistent associations across both diseases. Genetically predicted HSPA1B expression in classical monocytes was positively associated with COPD and coronary atherosclerosis, whereas expression in non‐classical monocytes showed inverse associations. NK cell‐specific HSPA1B expression was also positively associated with COPD.

**Conclusion:**

COPD and atherosclerosis share myeloid‐centered immune features, and HSPA1B may represent a cell‐state‐dependent immune signal linking pulmonary and vascular inflammation.

Chronic obstructive pulmonary disease (COPD) and atherosclerosis frequently coexist and together contribute substantially to morbidity and mortality [1]. Although systemic inflammation has long been considered a major biological bridge between pulmonary and vascular injury [2], the shared immune‐cell programs underlying this comorbidity remain insufficiently defined. To address this question, we integrated single‐cell RNA‐sequencing analyses of COPD and atherosclerotic lesions with cell‐type‐specific Mendelian randomization (MR) to identify shared immune signals across these two diseases.

Single‐cell profiling of the COPD dataset revealed a complex pulmonary microenvironment composed of epithelial, endothelial, stromal, and multiple immune populations, among which macrophages and monocytes displayed particularly prominent remodeling (Figure 1A–C). Grouped cell annotation and cell composition analysis suggested substantial myeloid reshaping in COPD, while marker‐gene heatmaps confirmed distinct macrophage‐ and monocyte‐associated transcriptional programs (Figure 1A–C). The detailed workflow of quality control, dimensionality reduction, clustering, and annotation is shown in Supporting Information S1: Figure S1. Notably, cell–cell communication analyses further demonstrated that macrophages occupied a central position in the COPD signaling network, as supported by both interaction number and interaction strength (Figure 1G,H). Representative ligand–receptor interactions in COPD are further shown in Supporting Information S1: Figure S3A. Together, these findings indicate that COPD is characterized by a macrophage/monocyte‐centered inflammatory architecture, in line with previous single‐cell observations that immune and epithelial remodeling are central features of COPD lungs [3].

**Figure 1:**
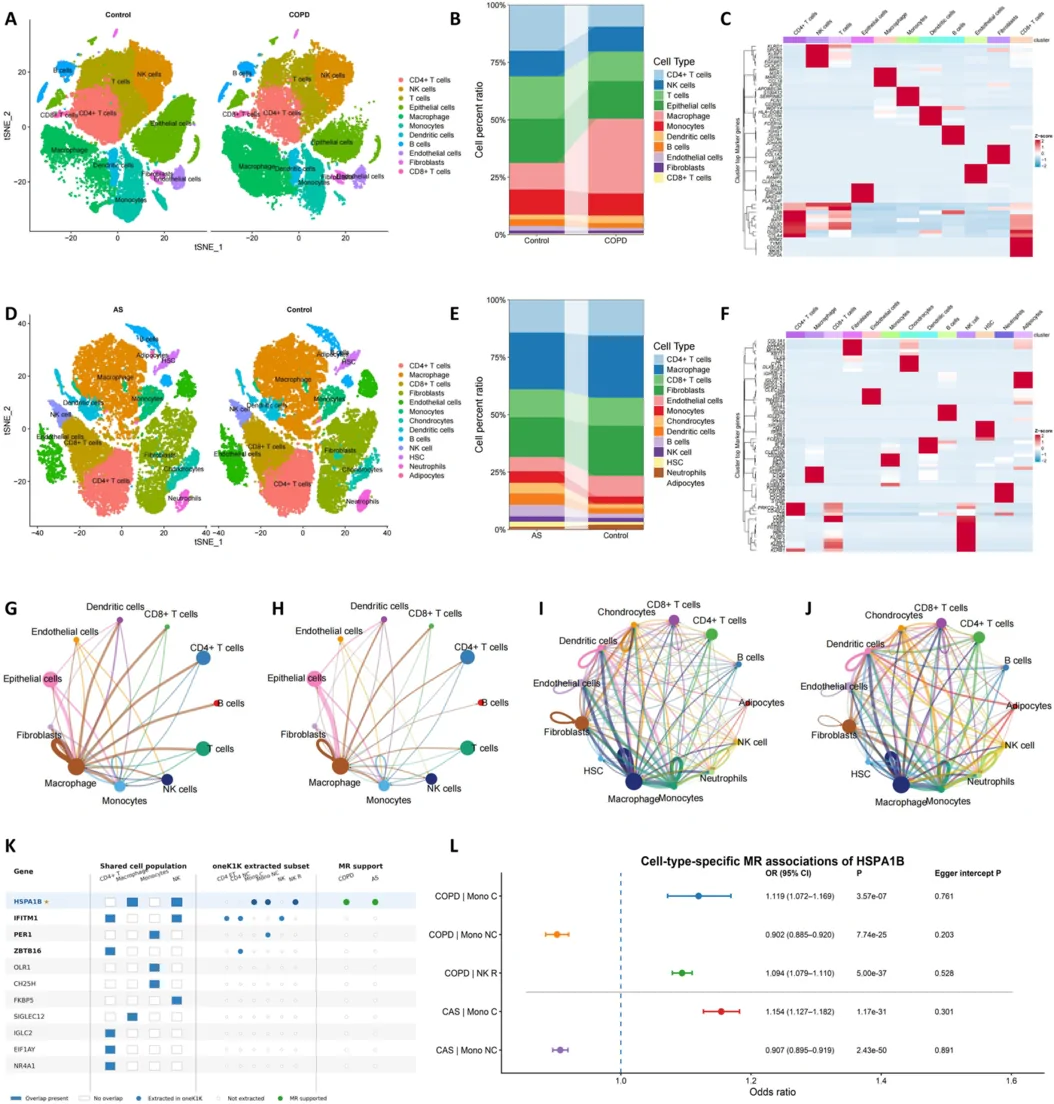
Integrative single‐cell and cell‐type‐specific genetic analyses identify HSPA1B as a shared immune signal linking COPD and coronary atherosclerosis. (A–C) Single‐cell analysis of the COPD dataset showing grouped cell annotation (A), relative cell composition (B), and representative marker genes (C). (D–F) Single‐cell analysis of the atherosclerosis dataset showing grouped cell annotation (D), relative cell composition (E), and representative marker genes (F). (G–J) Cell–cell communication networks in COPD (G, H) and atherosclerosis (I, J) based on interaction number (G, I) and interaction strength (H, J), highlighting macrophage/monocyte‐centered communication in both diseases. (K) Summary matrix of overlapping upregulated genes across matched immune cell populations in COPD and atherosclerosis, together with oneK1K extraction status. (L) Forest plot of cell‐type‐specific Mendelian randomization results for HSPA1B, showing positive associations in classical monocytes with both COPD and coronary atherosclerosis, inverse associations in non‐classical monocytes, and a positive association in NK cells with COPD.

Parallel analyses of the atherosclerotic single‐cell dataset identified a similarly immune‐rich lesional landscape, again dominated by myeloid populations (Figure 1D–F). Grouped cell annotation, relative cell composition, and marker‐gene heatmaps consistently supported substantial macrophage and monocyte involvement in atherosclerotic plaques (Figure 1D–F). The corresponding analytical workflow is shown in Supporting Information S1: Figure S2. Moreover, cell–cell communication analysis of atherosclerotic lesions highlighted macrophages as major signaling hubs in both count‐ and weight‐based networks (Figure 1I,J), while representative ligand–receptor pairs are shown in Supporting Information S1: Figure S3B. These results suggest that COPD and atherosclerosis share a common macrophage/monocyte‐centered immune organization, consistent with prior single‐cell studies showing marked myeloid heterogeneity in human atherosclerotic plaques [4].

Despite this broad cellular convergence, cross‐disease comparison of matched immune‐cell populations yielded only a limited number of overlapping upregulated genes (Figure 1K; Supporting Information S1: Table S2). Differentially expressed genes were identified using the Wilcoxon rank‐sum test with Benjamini–Hochberg correction. Upregulated genes were defined as those with adjusted *p* < 0.05 and log2 fold change >0.25. Specifically, overlapping upregulated genes were identified in CD4+ T cells (*IFITM1*, *IGLC2*, *EIF1AY*, *NR4A1*, and *ZBTB16*), macrophages (*HSPA1B* and SIGLEC12), monocytes (*OLR1*, *PER1*, and *CH25H*), and NK cells (*IFITM1*, *FKBP5*, and *HSPA1B*) (Figure 1K; Supporting Information S1: Table S2). This relatively restricted overlap suggests that although the two diseases share similar immune‐cell frameworks, their local tissue microenvironments impose substantial transcriptional divergence. Nevertheless, the retained intersecting genes may represent conserved immune nodes across pulmonary and vascular inflammation. Querying the oneK1K immune‐cell‐specific sc‐eQTL resource further showed that only a subset of these overlapping upregulated genes could be successfully extracted (Supporting Information S1: Table S3). Because macrophage‐specific sc‐eQTLs were unavailable in oneK1K, circulating monocyte subsets were used as genetically accessible proxies for macrophage‐related myeloid programs, based on their shared myeloid lineage and the capacity of circulating monocytes to differentiate into macrophage‐like cells after recruitment to inflamed tissues. In the oneK1K peripheral blood dataset, classical and non‐classical monocytes corresponded to CD14‐dominant and CD16/FCGR3A‐dominant monocyte subsets, respectively, consistent with the canonical CD14^high^CD16^low/^^−^ and CD14^low^CD16^high^ phenotypes.

We next performed cell‐type‐specific MR analyses using oneK1K sc‐eQTLs as exposures and FinnGen GWAS summary statistics for COPD and coronary atherosclerosis as outcomes. Among all evaluated candidates, *HSPA1B* emerged as the only gene showing robust and reproducible associations across both diseases (Figure 1L; Supporting Information S1: Table S4). Genetically predicted *HSPA1B* expression in classical monocytes was positively associated with COPD risk (OR = 1.119, 95% CI 1.072–1.169, *p* = 3.57 × 10^−7^) and coronary atherosclerosis risk (OR = 1.154, 95% CI 1.127–1.182, *p* = 1.17 × 10^−31^) (Figure 1L; Supporting Information S1: Table S4). In contrast, *HSPA1B* expression in non‐classical monocytes was inversely associated with COPD (OR = 0.902, 95% CI 0.885–0.920, *p* = 7.74 × 10^−25^) and coronary atherosclerosis (OR = 0.907, 95% CI 0.895–0.919, *p* = 2.43 × 10^−50^) (Figure 1L; Supporting Information S1: Table S4). In addition, NK cell‐restricted *HSPA1B* expression was positively associated with COPD risk (OR = 1.094, 95% CI 1.079–1.110, *p* = 5.00 × 10^−37^) (Figure 1L; Supporting Information S1: Table S4). Sensitivity analyses based on the MR‐Egger intercept test did not suggest marked directional pleiotropy (Supporting Information S1: Table S5). These findings identify *HSPA1B* as the lead cross‐disease immune signal emerging from our integrative framework.

Our study provides two major insights. First, COPD and atherosclerosis share a common myeloid‐centered inflammatory organization, particularly characterized by macrophage‐centered communication networks and monocyte involvement. Second, *HSPA1B* appears to exert cell‐state‐dependent effects across disease‐relevant immune subsets. *HSPA1B* encodes a stress‐inducible HSP70‐family molecular chaperone involved in proteostasis and cellular stress adaptation, and HSP70‐related pathways have been widely implicated in inflammatory regulation and immune‐cell stress responses [5]. The opposing associations of HSPA1B across monocyte subsets suggest that its biological effects may depend on the underlying myeloid cell state. Classical monocytes are preferentially recruited during sustained inflammation and can contribute to cytokine production and the generation of inflammatory macrophage populations, processes relevant to both COPD and atherosclerotic vascular injury. In this context, higher HSPA1B expression may reflect or contribute to cellular stress adaptation and persistence within a pro‐inflammatory environment. By contrast, non‐classical monocytes are more closely associated with endothelial surveillance, vascular homeostasis, and resolution‐related functions. Their inverse genetic association, therefore, raises the possibility that HSPA1B participates in a more homeostatic stress‐response program in this cellular state. Together, these divergent effects suggest that HSPA1B may contribute to shared pulmonary–vascular inflammation in a monocyte‐state‐dependent manner rather than exerting a uniform effect across the myeloid compartment.

Several limitations should also be acknowledged. The single‐cell discovery analyses were derived from distinct tissue environments, and the genetic validation relied on circulating immune‐cell sc‐eQTLs rather than lesion‐resident cells; thus, the monocyte‐based associations should be interpreted as reflecting shared myeloid regulation rather than macrophage‐specific effects. Although MR‐Egger analyses did not indicate marked directional pleiotropy, residual LD‐related confounding at the HSPA1B locus cannot be fully excluded without formal colocalization analysis. In addition, this study was computational and genetic in nature, and further mechanistic experiments will be required to define how HSPA1B regulates myeloid and NK‐cell function. Despite these limitations, our data support a model in which COPD and coronary atherosclerosis are linked by a shared macrophage/monocyte‐centered immune architecture, with *HSPA1B* representing a promising cell‐type‐specific signal at the intersection of pulmonary and vascular inflammation.

## Author Contributions

Yangxin Zhao, Fangzheng Cao, Houwen Zhang, and Yu Liang contributed equally to this work and share first authorship. They performed the analyses, prepared the figures, interpreted the data, and drafted the manuscript. You Wu conceived and supervised the study, revised the manuscript, and approved the final version for submission. All authors read and approved the final manuscript.

## Ethics Statement

This study was based exclusively on publicly available de‐identified datasets and did not require additional ethical approval.

## Conflicts of Interest

The authors declare no conflicts of interest.

## Supporting information


Supporting File 1

Supporting File 2

## Data Availability

The public datasets used in this study are summarized in Supporting Information S1: Table S1. The COPD single‐cell RNA‐sequencing dataset was obtained from the GEO database under accession GSE279570, and the atherosclerosis single‐cell RNA‐sequencing dataset was obtained from GEO under accession GSE253903. Immune‐cell‐specific sc‐eQTL data were derived from the oneK1K resource. GWAS summary statistics for COPD and coronary atherosclerosis were obtained from the FinnGen database. All data analyzed in this study are publicly available from the corresponding repositories.
