# Supplementary material for: Cell‐Type‐Specific HSPA1B Expression Links COPD and Atherosclerosis
Source: Immun Inflamm Dis. 2026 Sep 29;14(9):e70530. doi: 10.1002/iid3.70530 (PMC13621231; doi:10.1002/iid3.70530)
Supplement: Supplementary file 1 [file IID3-14-e70530-s001.pdf]

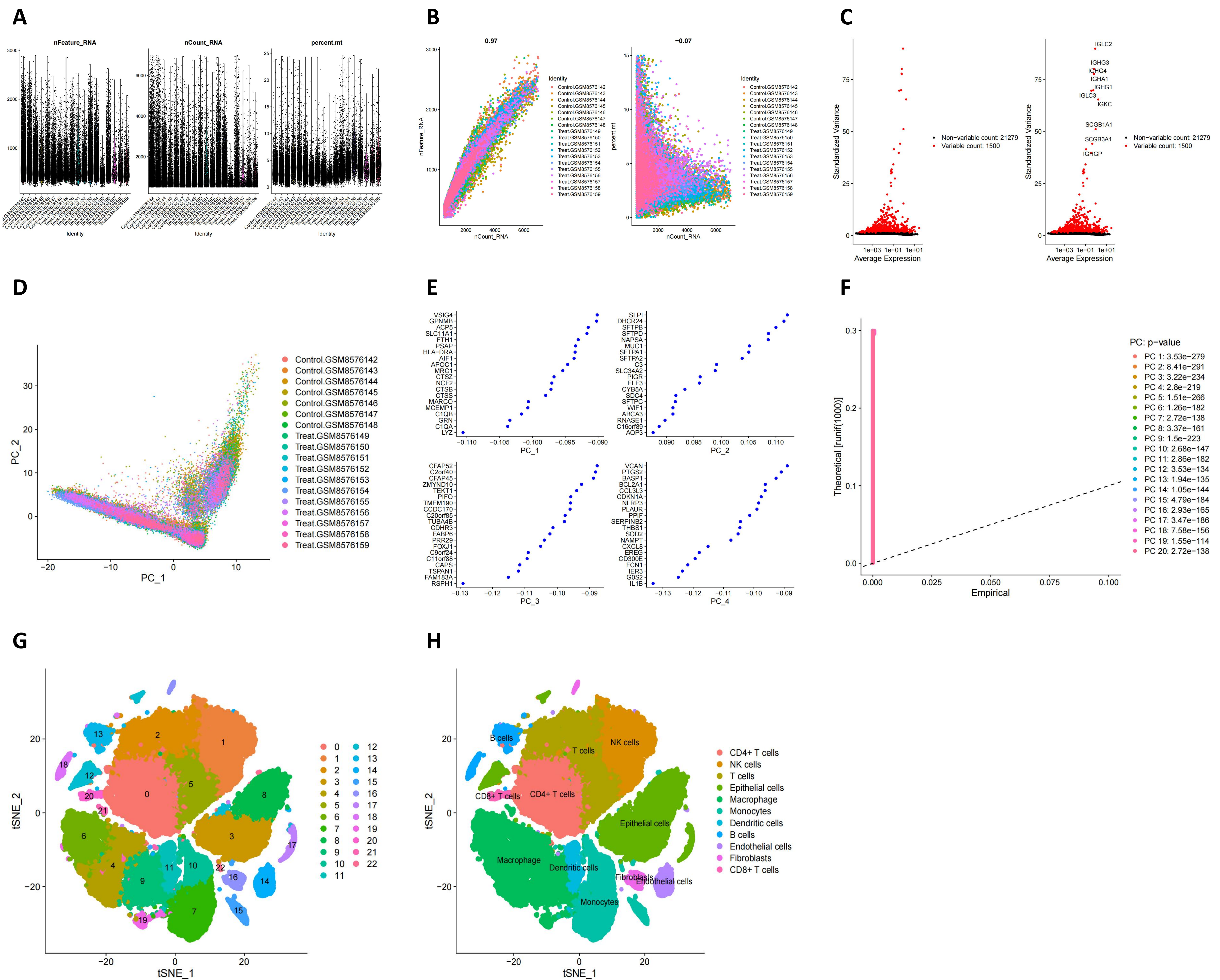

Supplementary Figure 1. Workflow of quality control, dimensionality reduction, clustering, and cell annotation for the COPD single-cell RNA-sequencing dataset.

A, Violin plots of quality-control metrics. **B**, Correlation plots of nCount_RNA, nFeature_RNA, and percent.mt. **C**, Highly variable genes. **D**, PCA plot. **E**, Representative PCA loading genes. **F**, JackStraw plot. **G**, t-SNE clustering plot. **H**, Annotated major cell populations.

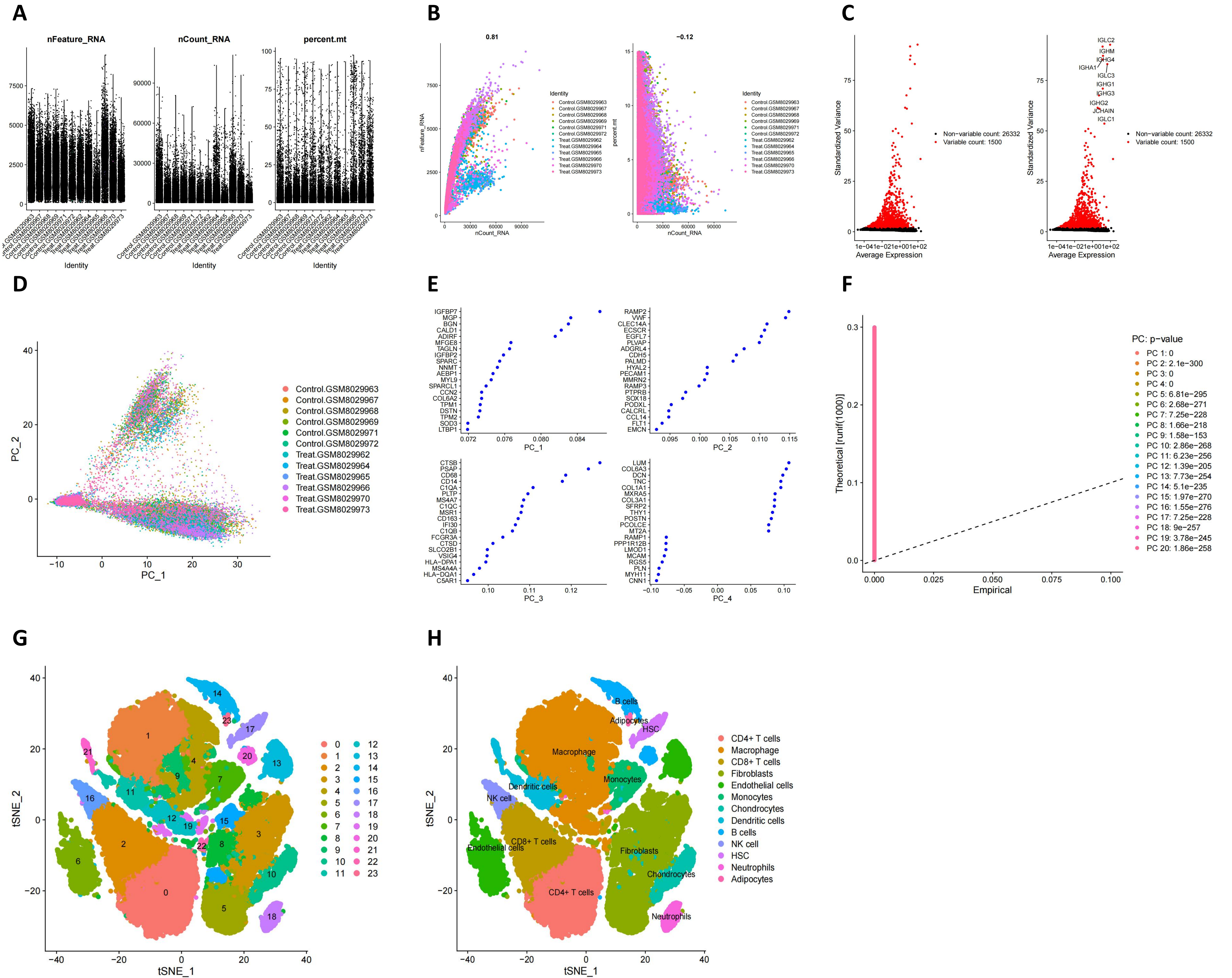

Supplementary Figure 2. Workflow of quality control, dimensionality reduction, clustering, and cell annotation for the AS single-cell RNA-sequencing dataset.

A, Violin plots of quality-control metrics. **B**, Correlation plots of nCount_RNA, nFeature_RNA, and percent.mt. **C**, Highly variable genes. **D**, PCA plot. **E**, Representative PCA loading genes. **F**, JackStraw plot. **G**, t-SNE clustering plot. **H**, Annotated major cell populations.

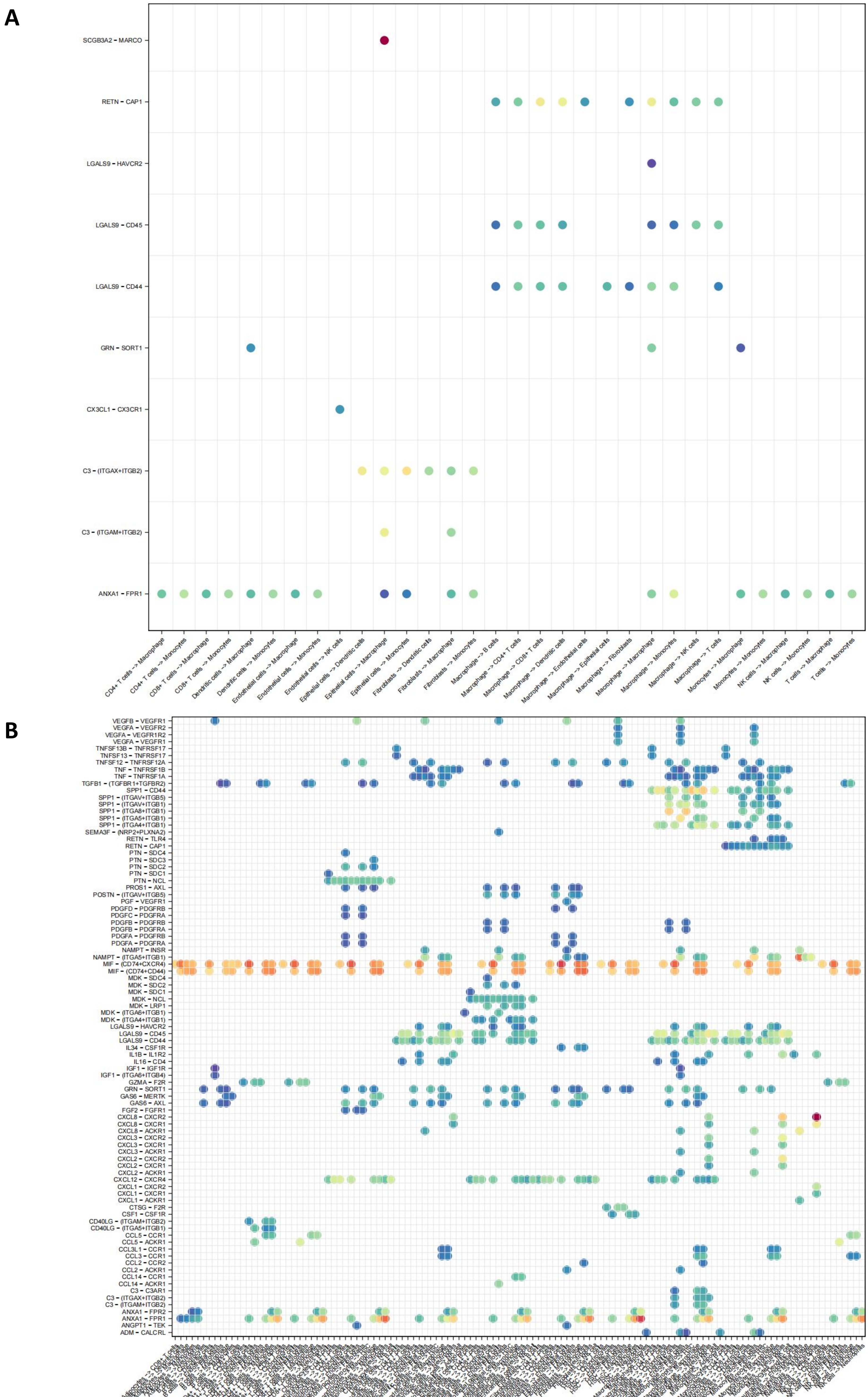

Supplementary Figure 3. Bubble plots of representative ligand–receptor interactions in COPD and AS.

A, Representative ligand–receptor interactions in COPD. **B**, Representative ligand–receptor interactions in AS.
